# A pharmacovigilance study of chronic kidney disease in diabetes mellitus patients with statin treatment by using the US Food and Drug Administration adverse event reporting system

**DOI:** 10.3389/fphar.2024.1363501

**Published:** 2024-06-21

**Authors:** Jingyi Zhang, Yuting Guo, Chunyan Wei, Yu Yan, Huifang Shan, Bin Wu, Fengbo Wu

**Affiliations:** ^1^ Department of Pharmacy, West China Hospital, Sichuan University, Chengdu, China; ^2^ West China School of Pharmacy, Sichuan University, Chengdu, China

**Keywords:** statins, diabetes mellitus, chronic kidney disease, FAERS, disproportionality analyses

## Abstract

**Background:**

Statins were regarded as a main medication for managing hypercholesterolemia. Administration of statin therapy could reduce the incidence of cardiovascular disease in individuals diagnosed with type 2 diabetes mellitus (DM), which was recognized by multipal clinical guidelines. But previous studies had conflicting results on whether the long-term use of statins could benefit the renal function in diabetic patients.

**Aim:**

To evaluate the association between statin treatment and Chronic Kidney Disease in DM patients.

**Methods:**

This is a retrospective disproportionality analysis and cohort study based on real-world data. All DM cases reported in US Food and Drug Administration adverse event reporting system (FAERS) between the first quarter of 2004 and the fourth quarter of 2022 were included. Disproportionality analyses were conducted by estimating the reporting odds ratio (ROR) and the information component (IC). We further compared the CKD odds ratio (OR) between the statins group and the other primary suspected drug group among the included diabetes mellitus cases.

**Results:**

We finally included 593647 DM cases from FAERS, 5113 (5.31%) CKD cases in the statins group and 8810 (1.77%) CKD cases in the control group. Data analysis showed that the statins group showed a significant CKD signal (ROR: 3.11, 95% CI: 3.00–3.22; IC: 1.18, 95% CI: 1.07–1.29). In case group with two or more statins treatment history, the CKD signal was even stronger (ROR: 19.56, 95% CI: 18.10–21.13; IC: 3.70, 95% CI:3.44–3.93) compared with cases with one statin treatment history.

**Conclusion:**

The impact of statin therapy on the progression of renal disease in individuals diagnosed with type 2 diabetes mellitus (DM) remains inconclusive. After data mining on the current FAERS dataset, we discovered significant signals between statin treatment and CKD in diabetic patients. Furthermore, the incidence rate of CKD was higher among DM patients who used statins compared to those who did not.

## 1 Introduction

Statins (hydroxymethylglutaryl-Coenzyme A reductase inhibitors) are the most prescribed drugs in clinical practice, which was effective in lowering LDL cholesterol (Blais et al., 2021; Zhou et al., 2023). As has long been recognized, patients diagnosed with type 2 diabetes are more likely to develop hyperlipidemia (McGarry, 2002; Huang et al., 2023). Several studies shown that dyslipidemia was one of the risk factors for abnormal renal function in diabetic patients, leading to a gradual decline in renal function, associated with proteinuria and decreased glomerular filtration rate (GFR) (Chen and Tseng, 2013; Collins et al., 2016; Cai et al., 2021; DeFronzo et al., 2021; Kelsey et al., 2022; Zhou et al., 2023). Therefore, strict management of blood lipid levels becomes crucial for patients with type 2 diabetes.

The impact of lipid-lowering treatment on the progression of kidney disease in individuals diagnosed with type 2 diabetes mellitus remains uncertain. Multiple studies indicated that statins might possess protective properties against oxidative stress caused by diabetes and injury to the podocytes in the renal system (Vlad et al., 2017; Aktay et al., 2019). A multicenter, retrospective cohort study suggested that statin initiation was associated with a lower risk of kidney disease development, particularly in those with intensive LDL-C control (Zhou et al., 2023). However, there were other research findings suggested that the use of statin therapy might result in alterations in glucose metabolism and affect glycemic regulation among individuals with diabetes, potentially increasing the likelihood of microvascular complications in patients receiving statin treatment (Holman et al., 2009; Koh et al., 2010; Liew et al., 2014; Abbasi and Reaven, 2015; Erlandson et al., 2015; Thomas et al., 2015; Mansi et al., 2021). Abnormal renal function was also a prevalent microvascular complication observed in patients diagnosed with type 2 diabetes. Furthermore, a research study demonstrated that the prolonged utilization of statins may lead to ectopic fat accumulation, resulting in inflammation and fibrosis. Ultimately, this process can expedite the advancement of diabetic nephropathy (Huang et al., 2023). Additional studies conducted on population level have also provided evidence in line with the aforementioned perspective, indicated that the utilization of statins did not offer a risk reduction for kidney disease and could potentially lead to adverse effects among individuals diagnosed with diabetes (Bangalore et al., 2014; Nielsen and Nordestgaard, 2014).

As mentioned above, previous studies showed conflicting results on whether statins benefit kidney function in people with diabetes. Therefore, we hoped to explore whether statin use improves the reporting rate of CKD in patients with diabetes through real-world data from the FDA Adverse Event Reporting System (FAERS) database. Expected to provide reference for the safety of drug use in clinical practice, and provide guidance for the pharmaceutical care of clinicians and pharmacists.

## 2 Materials and methods

### 2.1 Materials

This was a retrospective, observational pharmacovigilance study designed to analyze the association between CKD events and statins use in patients with DM. The original data were obtained from adverse reports in the FAERS database from the first quarter of 2004 to the fourth quarter of 2022.

The FAERS dataset comprised of seven distinct data Modules: patient demographic and administrative information (DEMO), drug details (DRUG), patient outcomes (OUTC), adverse events (REAC), report sources (RPSR), indications for drug administration (INDI), and therapy start dates and end dates for reported drugs (THER). In accordance with FDA recommendations, a clean, drug-mapped, de-duplicated version of the FAERS data was extracted (Wu et al., 2022). If the CASEIDs (a number used to identify a FAERS case) were the same, the latest FDA_DT (date FDA received the case) was selected. If the CASEID and FDA_DT were the same, the higher PRIMARYID (a unique number for identifying a FAERS report) was selected. Subsequently, we used the MedEx 1.3.8 software to standardize different names of the same drug into the “generic name” (Wei et al., 2022). The REAC module and INDI module were both coded by MedDRA preferred terms (Wu et al., 2019). After indication identification, we eliminated cases with CKD cases reported in the INDI module.

### 2.2 Methods

#### 2.2.1 Target case identification

The classification and standardization of disease diagnoses, adverse reactions in the FAERS database are referenced by the Medical Dictionary of Regulatory Activities (MedDRA), and each report is coded using preferred terms (PTs). In MedDRA terminology. Different PTs could be collected to define a disease diagnoses and specific adverse reaction through Standardized MedDRA Queries (SMQs) (Tian et al., 2022).

According to the Medical Dictionary for Regularly Activities (MedDRA) and Standardized MedDRA Queries (SMQs) version 23.1. We identified DM cases using SMQ coded 20000041 narrow searching, which included 35 preferred terms (PTs), shown in Supplementary Table S1. We identified CKD cases using SMQ coded 20000213 narrow searching, which included 42 PTs, shown in Supplementary Table S2. Duplicate records were removed in case one case was reported under multiple PTs of the same SMQ.

#### 2.2.2 Target drugs identification

The DRUG table contains drug names that may be documented in various formats, such as generic names, synonymous names, brand names, or abbreviations. To accurately identify target drugs, we utilized MedEx software (MedEx UIMA 1.3.8, Vanderbilt University, USA) to standardize different variations of the same drug into a “generic name”. We attempted to identify eight single component statins according to the WHO Anatomical Therapeutic Chemical (ATC) classification (ATC code: QC01AA) from the local FAERS database, shown in shown in Supplementary Table S3. Drugs in the DRUG table were classified into primary suspected (PS) drugs, secondary suspected (SS) drugs, concomitant (C) drugs, and interacting (I) drugs. The current study only included PS drugs.

#### 2.2.3 Statistical analysis

##### 2.2.3.1 Signal analysis

We first analyzed the chronic kidney disease (CKD) disproportionate signal by the algorithms of reporting odds ratios (ROR) and the information components (IC). We managed the FAERS dataset in local use through Microsoft SQL Server 2017 software. The characteristics of CKD cases and non-CKD cases with target drugs were collected, including age, sex, report year, report country, identity of reporter (health professionals or non-health professionals). Algorithms of the reporting odds ratio (ROR) and information component (IC) were used to detect the association between CKD events and target drugs (Li et al., 2008). Microsoft Excel 2013 (Microsoft, Redmond, Washington, USA) were used to calculate the value of ROR and IC, including the 95% confidence interval (95% CI) of them. For ROR, the significant association was detected when the case number was ≥3 and the lower limit of the 95% CI was >1. For the IC method, if IC > 0 and the lower limit of 95% CI was >0, the signals were considered significant (Wei et al., 2023b). The ROR value was used as the primary assay, and the IC value was used as the confirmation method. The CKD events were considered to be associated with the target drug only when both the ROR and the IC methods met their threshold.

##### 2.2.3.2 Comparison between groups

We further compared the odds ratio (OR) for chronic kidney disease (CKD) between the group taking statins and the group taking other primary suspected drugs among the included cases of diabetes mellitus.

## 3 Results

### 3.1 Characteristics analysis

**FIGURE 1:**
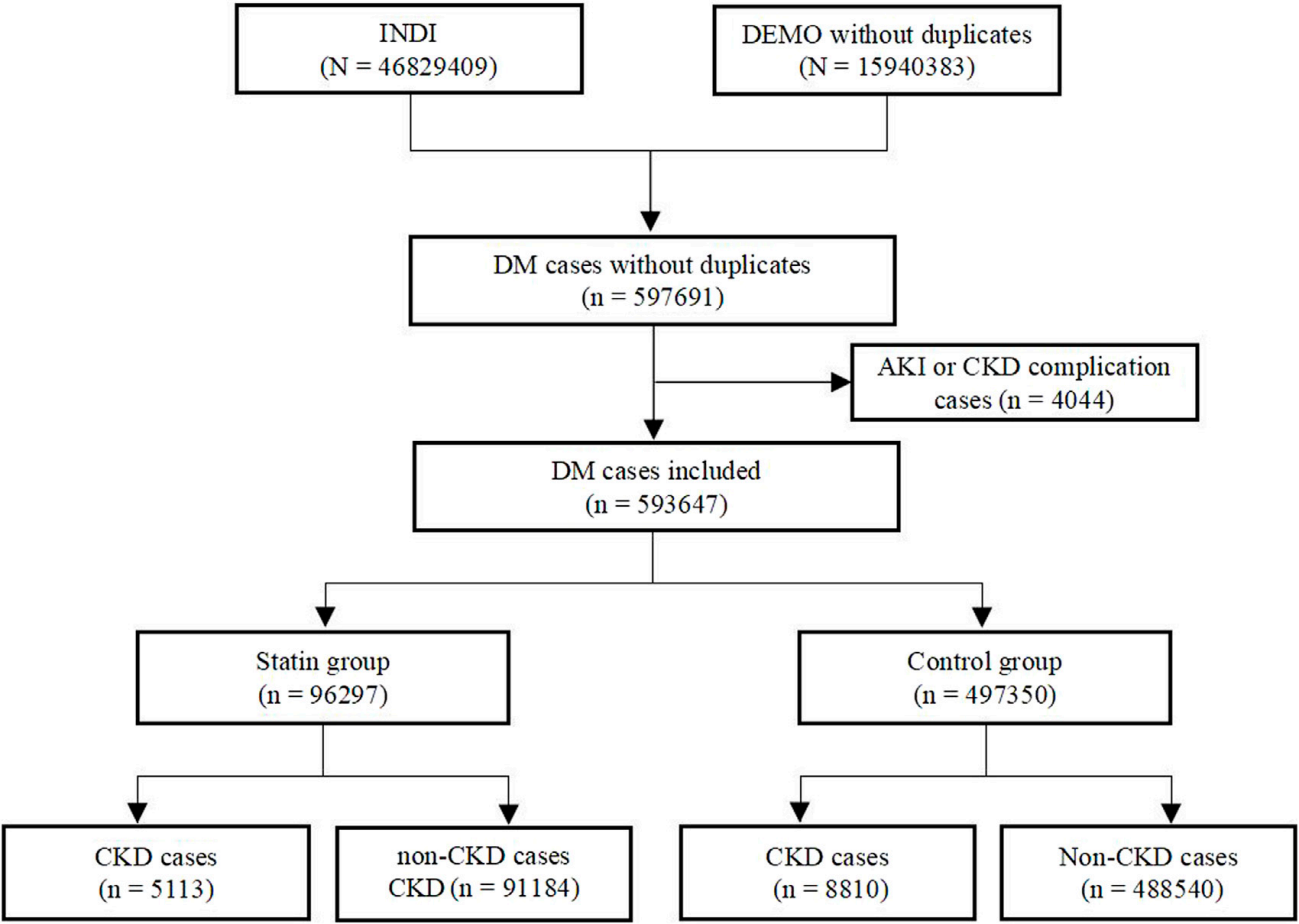
Flow chart of the identification of CKD cases in DM patients treated with statins from the FAERS database.

We identified a total of 597,691 adverse events in patients with DM from the FAERS database from January 2004 to December 2022. Reports of complications related to AKI and CKD were excluded, resulting in the inclusion of 593,647 cases for disproportionality analysis. Among these, 96,297 cases were assigned to the statins group and 497,350 cases to the control group. We further identified 5113 statins cases reported with CKD events and 91184 cases for the non-CKD events. In the control group, a total of 8,810 cases were reported for statin-related CKD events, while non-CKD events were reported in 488,540 cases. The details of the case identification were shown in Figure 1.

We summarized the clinical characteristics of patients, these features were described in Table 1. The number of drug-related CKD events reported in DM was higher in the control group than in the statins group. Among the age-related cases, the proportion of CKD events in DM was higher in the 18–65 group than in other age groups, while the proportion of CKD events in diabetic patients who did not use statins was higher in the over 65 age group than in other age groups. The number of reported CKD cases in DM patients using statins was almost the same in female as in male, the reported number were 2240 and 2230, respectively. Health professionals reported 36.9% of CKD cases in the statins group. In the statins group, North America (73.60%) reported the most cases of CKD events, followed by Europe (19.20%) and Asian (2.40%). CKD events reported in 2019 were the highest in both statins and control group, accounting for 24.40% and 11.60%, respectively (Table 1).

**TABLE 1:** Primary characteristics of CKD events in DM patients from FAERS.

Characteristics	Statins group		Control group	
	CKD cases/n (%)	Non-CKD cases/n (%)	CKD cases/n (%)	Non-CKD cases/n (%)
DM case	5113 (100.00)	91184 (100.00)	8810 (100.00)	488540 (100.00)
Age group				
<18 yr	58 (1.10)	1223 (1.30)	261 (3.00)	13284 (2.70)
18–65 yr	2101 (41.10)	34643 (38.00)	3028 (34.40)	175656 (36.00)
≥65 yr	1796 (35.10)	42070 (46.10)	3185 (36.20)	144340 (29.50)
unknown	1158 (22.60)	13248 (14.50)	2336 (26.50)	155260 (31.80)
Sex				
Female	2240 (43.80)	42989 (47.10)	4171 (47.30)	243721 (49.90)
Male	2230 (43.60)	45046 (49.40)	3957 (44.90)	201438 (41.20)
unknown	643 (12.60)	3149 (3.50)	682 (7.70)	43381 (8.90)
Reporter				
HP	1889 (36.90)	47987 (52.60)	3848 (43.70)	169050 (34.60)
non-HP	1691 (33.10)	37497 (41.10)	4069 (46.20)	303600 (62.10)
unknown	1533 (30.00)	5700 (6.30)	893 (10.10)	15890 (3.30)
Report region				
Africa	22 (0.40)	563 (0.60)	88 (1.00)	2900 (0.60)
Asian	122 (2.40)	7028 (7.70)	514 (5.80)	32350 (6.60)
Europe	980 (19.20)	23851 (26.20)	2192 (24.90)	54684 (11.20)
North America	3761 (73.60)	53653 (58.80)	5368 (60.90)	380646 (77.90)
Oceania	24 (0.50)	830 (0.90)	69 (0.80)	2145 (0.40)
South America	114 (2.20)	3501 (3.80)	280 (3.20)	10357 (2.10)
unknown	90 (1.80)	1758 (1.90)	299 (3.40)	5458 (1.10)
Report year				
2004–2008	318 (6.20)	8900 (9.70)	972 (11.10)	49173 (10.00)
2009–2013	646 (12.80)	19451 (21.30)	2166 (24.60)	82830 (17.00)
2014–2018	1194 (23.40)	37000 (40.60)	2816 (32.00)	225963 (46.30)
2019–2022	2955 (57.70)	25833 (28.40)	2856 (32.40)	13.574 (26.80)

### 3.2 CKD signal detection in statins group and control group in DM from FAERS

Firstly, we detected the chronic kidney disease (CKD) signal of both the statins group and control group in diabetic cases, using all other cases detected in the FDA Adverse Event Reporting System (FAERS) as background data. Notably, significant signals were observed in both groups The ROR values were (ROR: 4.48, 95% CI: 4.35–4.61; IC: 2.07, 95% CI: 1.98–2.17) for the statins group and (ROR: 1.43, 95% CI: 1.40–1.46; IC: 0.49, 95% CI: 0.42–0.56) for the control group. The lower limits of the 95% confidence intervals for all ROR values were greater than 1, and the lower limits of all IC values were greater than zero.

Secondly, we detected the CKD signal in both the statins group and control group among DM cases, using other cases in the DM patient population as background. Only the statins group showed a significant signal, with ROR values of (ROR: 3.11, 95% CI: 3.00–3.22; IC: 1.18, 95% CI: 1.07–1.29), while the control group had ROR values of (ROR: 0.32, 95% CI: 0.31–0.33; IC:–0.40, 95% CI:–0.49–0.32). These results indicated a higher CKD constituent ratio in DM patients than that of the background population and suggested that the CKD constituent ratio of DM patients was higher than that of background population.

We detected a significantly stronger signals among individuals with a history of using two or more statins compared to those who had only used one statin. The results for two or more statins history were (ROR: 19.56, 95% CI: 18.10–21.13; IC: 3.70, 95% CI:3.44–3.93), while the value of the one statin histoty group were (ROR: 2.34, 95% CI: 2.25–2.42; IC: 0.92, 95% CI: 0.81–1.04).

**FIGURE 2:**
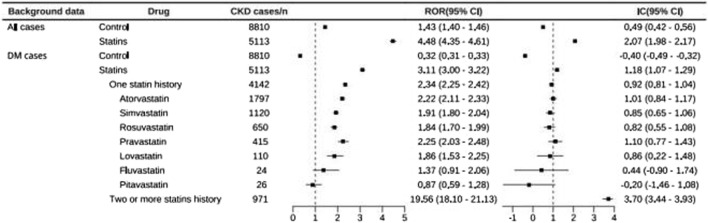
CKD signal detection in statins group and control group in DM patients from FAERS.

In the data from the group with a history of one statin, pravastatin (ROR: 2.25, 95% CI: 2.03–2.48; IC: 1.10, 95% CI:0.77–1.43) showed the strongest signal, followed by atorvastatin (ROR: 2.22, 95% CI: 2.11–2.33; IC: 1.01, 95% CI:0.84–1.17), simvastatin (ROR: 1.91, 95% CI: 1.80–2.04; IC: 0.85, 95% CI:0.65–1.06), lovastatin (ROR: 1.86, 95% CI: 1.53–2.25; IC: 0.86, 95% CI:0.22–1.48) and rosuvastatin (ROR: 1.84, 95% CI: 1.70–1.99; IC: 0.82, 95% CI:0.55–1.08) in descending order of ROR value. Whether the Fluvastatin (ROR: 1.37, 95% CI: 0.91–2.06; IC: 0.44, 95% CI: −0.90–1.74) and Pitavastatin (ROR:0.87, 95% CI: 0.59–1.28; IC: −0.20, 95% CI: −1.46–1.08) increase CKD needed further verification due to the lower limit of 95% CI for IC being <0 (Figure 2).

### 3.3 CKD comparison between statins group and control group

We calculated the proportion of CKD events separately for each statin in patients with DM compared to controls. The results showed a higher proportion of CKD occurrence in the statins group than in the control group among DM cases from FAERS. Except for pitavastatin with 95% CI for OR (0.78–1.71), the CKD constituent ratios of other statins were statistically different from those of the control group (Table 2).

**TABLE 2:** CKD comparison between Statins group and control group.

Drug	CKD cases/n	CKD proportion/%	Total cases/N	OR	95%CI for OR
Statins	5113	5.31	96297	3.11	(3.00–3.22)
Atorvastatin	1797	4.72	38103	2.74	(2.61–2.89)
Simvastatin	1120	4.23	26458	2.45	(2.30–2.61)
Rosuvastatin	650	4.14	15690	2.40	(2.21–2.60)
Pravastatin	415	5.04	8233	2.94	(2.66–3.26)
Lovastatin	110	4.26	2584	2.47	(2.03–2.99)
Pitavastatin	26	2.05	1271	1.16	(0.78–1.71)
Fluvastatin	24	3.19	753	1.83	(1.22–2.74)
Cerivastatin	0	0.00	753	0.00	-

Then, we analyzed the cases reported by health professionals. Compared to the data in Table 3, the ORs for all statins decreased, however, there were still statistically significant differences in the CKD constituent ratios of atorvastatin, simvastatin, rosuvastatin, pravastatin, and lovastatin compared to those of the control group. There was no significant difference in CKD constituent ratio between pitavastatin, fluvastatin and the control group (Table 3).

**TABLE 3:** CKD comparison between Statins group and control group with cases reported by health professionals.

Drug	CKD cases/n	CKD proportion/%	OR	95%CI for OR
Statins	1889	3.79	1.73	(1.64–1.83)
Atorvastatin	749	3.66	1.67	(1.54–1.81)
Simvastatin	513	3.81	1.74	(1.58–1.91)
Rosuvastatin	293	3.50	1.59	(1.41–1.80)
Pravastatin	147	3.65	1.67	(1.41–1.97)
Lovastatin	32	3.48	1.58	(1.11–2.26)
Pitavastatin	19	2.06	0.92	(0.58–1.45)
Fluvastatin	12	2.22	1.00	(0.56–1.77)
Cerivastatin	0	0.00	0.00	-

## 4 Discussion

Statins are one of the mainstays of treatment and widely used for lowering cholesterol (Gotto and Moon, 2012). Statins have demonstrated benefits in preventing coronary artery disease and reducing cardiovascular risk in patients with type 2 diabetes mellitus (DM) and hypertension, thus benefiting the majority of high-risk individuals (Endo, 1988; Blais et al., 2021; Collins et al., 2003; Adhyaru and Jacobson, 2018; O'Malley et al., 2020; Krane et al., 2016; Pedersen et al., 2004; Watanabe et al., 2006; Goldstein and Brown, 1990; Cholesterol Treatment Trialists’ Collaboration and Cholesterol Treatment Trialists’ Collaboration, 2024). For DM patients, elevated cholesterol level is one of the risk factors for diabetic nephropathy, so it is also very important for DM patients to strictly control the blood lipid level (Chen and Tseng, 2013; Collins et al., 2016; Cai et al., 2021; DeFronzo et al., 2021; Kelsey et al., 2022; Zhou et al., 2023). At present, some clinical guidelines recommend the use of statins for patients with special diabetes (Pearson et al., 2021; Marx et al., 2023). But, there are conflicting findings regarded the long-term renal benefits of statins in DM patients. Therefore, we wanted to analyze and evaluate the association between statin therapy and chronic kidney disease in DM patients by using data from FAERS. To the best of our knowledge, this is the first pharmacovigilance analysis of the association between statin use and chronic CKD in diabetic patients through FAERS.

Our current study analyzed the association between statins and the development of CKD in patients with DM using real-world data from the FAERS. The results of the study showed that, when compared to other cases of DM as background, CKD signals in the statins group and the control group in DM cases were calculated, the statins group showed an obvious signal, suggesting that the CKD composition ratio of DM patients using statins was higher than that of the background population, which was consistent with the results of the latest experimental study (Huang et al., 2023). But, the present finding contradicts the results of a recent multicenter retrospective study, which suggested that initiating statin therapy is significantly associated with a reduced risk of diabetic kidney disease (DKD) and improved renal function in patients diagnosed with type 2 diabetes (Zhou et al., 2023).

Diabetic kidney disease (DKD) was a prevalent microvascular complication observed in individuals diagnosed with DM, and it stand as the primary contributor to end-stage renal disease (Zagkotsis et al., 2018; Jairoun et al., 2023). The incidence of DKD can range from 20% to 40% among individuals with diabetes (de Boer and Group, 2014; Afkarian et al., 2016). The occurrence of diabetic kidney disease (DKD) is intricately associated with aberrant glucose metabolism, renal hemodynamic alterations, oxidative stress, genetic predisposition, and dysregulated lipid metabolism. Therefore, if statin therapy induced glycemic instability in diabetic patients or progresses to diabetes in pre-diabetic patients, the utilization of statins might theoretically augmented the susceptibility to renal impairment among individuals with diabetes.

The US Food and Drug Administration authorized a modification to the label in 2012. This revision included the addition of information stating that statins have been reported to cause increases in glycated haemoglobin (HbA1c) and fasting glucose levels (Force et al., 2022). Similarly, the European Medicines Agency acknowledged the heightened risk of developing diabetes among individuals using statins who are already at risk for this condition. An individual participant data meta-analysis published in 2024 suggested that statins cause a moderate dose-dependent increase in new diagnoses of diabetes that is consistent with a small upwards shift in glycaemia, with the majority of new diagnoses of diabetes occurring in people with baseline glycaemic markers that are close to the diagnostic threshold for diabetes (Cholesterol Treatment Trialists’ Collaboration and Cholesterol Treatment Trialists’ Collaboration, 2024).

Refractory glycemic control in diabetic patients receiving statin therapy may exacerbate renal dysfunction. As we knew, high blood glucose levels make endothelial cells mitochondrial oxidative stress, abnormal activation or inhibition of the downstream signaling pathways (hexose pathway, end products (AGEs) synthesis pathway, sorbitol pathway and protein kinase C pathway, etc.), finally through inflammation and oxidative stress cause endothelial cell damage, leading to impaired renal function (Alicic et al., 2017; Wu et al., 2023). What are the risk factors for new onset diabetes treated with statins? An updated meta-analysis including randomized trials showed that statin therapy could increase the risk of new-onset diabetes, especially with high dose statin (Cholesterol Treatment Trialists’ Collaboration and Cholesterol Treatment Trialists’ Collaboration, 2024). The second risk factor that contributed to the development of type 2 diabetes include abnormalities in fasting glucose levels, metabolic syndrome, and being overweight (Currie et al., 2013; Ahmadizar et al., 2019). The presence of fatty liver or increased epicardial fat thickness has been associated with a threefold higher risk of developing diabetes compared to individuals with minimal hepatic steatosis (Shah et al., 2015; Kang et al., 2018; Anastasiou et al., 2023). In trials involving participants aged over 65, a meta-analysis revealed a more pronounced correlation between statins and the likelihood of developing diabetes compared to trials with younger participants (Sattar et al., 2010). Although statin therapy might be associated with an elevated risk of new-onset diabetes or blood sugar disorders in individuals with diabetes, when considered the benefits of statin therapy for preventing and managing cardiovascular complications in this population, current evidence does not support discontinuation of statins. Instead, it is recommended to closely monitor blood glucose levels during treatment to mitigate potential complications arising from glycemic instability, including renal impairment (Cholesterol Treatment Trialists’ Collaboration and Cholesterol Treatment Trialists’ Collaboration, 2024).

Furthermore, our study showed that two or more statins history group exhibited stronger signals than the single-agent regimen. However, it was worth noting that the utilization of multiple statins in clinical practice was uncommon and typically limited to patients requiring a switch in statin therapy due to intolerance (Wei et al., 2023a). Due to the unavailability of information regarding concurrent or sequential use of multiple statins in the FAERS database, a comprehensive analysis and discussion of the data cannot be conducted. In cases where two or more statins are used simultaneously, it was generally considered a medication error that may elevate the risk of adverse drug reactions.

An observational, historical cohort study of type 2 diabetic Japanese patients with estimated glomerular filtration rate (eGFR) ≥30 mL/min/1.73 m^2^ suggested that lipophilic statins may exert a more pronounced detrimental impact on renal function (Hanai et al., 2017). Therefore, we compared the proportion of reported CKD in the statin and control groups of DM cases in the FAERs database. Our findings indicated that the proportion of CKD reports in the statin group was higher than the control group among DM cases from FAERs database. This difference was statistically significant for atorvastatin, simvastatin, rosuvastatin, pravastatin, lovastatin and fluvastatin but not pitavastatin. OR values for each statin decreased after excluding publicly reported and analyzing only health professional-reported cases; However, there was still a significant difference in the proportion of CKD components of atorvastatin, simvastatin, rosuvastatin, pravastatin, and lovastatin compared with the control group. We speculated that the lower odds reported by health professionals may be due to the lack of understanding of CKD by non-health professionals, because it requires a certain medical basis to make judgments, and health professionals’ judgments of CKD are more professional and rigorous. However, our findings were inconsistent with previous reports and require further research in this field.

Based on FAERS real-world data, our study revealed a significant association between CKD and statin treatment in diabetic patients. However, it was important to acknowledge the limitations of the study. Because there were some important limitations inherent to the use of the FAERS database. FAERS database itself had limitations. Firstly, the FAERS database was a spontaneous reporting system open to both health professionals and the public. Consequently, there might be instances of underreporting, overreporting, variations or missing information within the database leading to potential reporting bias that could be avoided. For example, health professionals were more concerned with serious and new adverse reactions, and may choose not to report minor adverse events and make more rigorous judgments about adverse reactions. Secondly, causality could not be inferred or determined because patient treatment information is often incomplete, including patient history and reported duration of drug use. We were unable to identify any other factors that might have influenced the results (Wei et al., 2023a). Finally, our study was unable to describe the mechanism behind the increased adverse effects. In subsequent studies, we anticipate more high-quality research to demonstrate the causal relationship between long-term statin use and CKD in diabetic patients, such as randomized controlled trials or case-control studies, and to provide more detailed explanations of its pathogenesis.

## 5 Conclusion

Through data mining from FAERS, we discovered a significant signals between statin therapy and the incidence of chronic kidney disease (CKD) events among DM patients. We speculated that statin use may contribute to the development of CKD in diabetic patients. However, this detected risk signal merely indicates a statistical association between drugs and ADE, serving as a basis for formulating hypotheses and guiding subsequent investigations. However, the establishment of a causal relationship necessitates further and more comprehensive research for validation.

## Data Availability

The datasets presented in this study can be found in online repositories. The names of the repository/repositories and accession number(s) can be found in the article/Supplementary Material.
