# Supplementary material for: A pharmacovigilance study of chronic kidney disease in diabetes mellitus patients with statin treatment by using the US Food and Drug Administration adverse event reporting system
Source: Front Pharmacol. 2024 Jun 21;15:1363501. doi: 10.3389/fphar.2024.1363501 (PMC11224537; doi:10.3389/fphar.2024.1363501)
Supplement: Supplementary file 1 [file Table1.DOCX]

**Supplement Table 1.** Preferred terms for identification of Diabetes Mellitus cases in FAERS

| **SMQ code** | **Preferred term code** | **Preferred term name** |
| --- | --- | --- |
| 20000041 | 10005557 | Blood glucose increased |
| 20000041 | 10012596 | Diabetes complicating pregnancy |
| 20000041 | 10012601 | Diabetes mellitus |
| 20000041 | 10012607 | Diabetes mellitus inadequate control |
| 20000041 | 10012631 | Diabetes with hyperosmolarity |
| 20000041 | 10012650 | Diabetic coma |
| 20000041 | 10012668 | Diabetic hyperglycaemic coma |
| 20000041 | 10012669 | Diabetic hyperosmolar coma |
| 20000041 | 10012671 | Diabetic ketoacidosis |
| 20000041 | 10012672 | Diabetic ketoacidotic hyperglycaemic coma |
| 20000041 | 10012673 | Diabetic ketosis |
| 20000041 | 10017395 | Fructosamine increased |
| 20000041 | 10018209 | Gestational diabetes |
| 20000041 | 10018429 | Glucose tolerance impaired |
| 20000041 | 10018430 | Glucose tolerance impaired in pregnancy |
| 20000041 | 10018473 | Glycosuria |
| 20000041 | 10018475 | Glycosuria during pregnancy |
| 20000041 | 10018478 | Glucose urine present |
| 20000041 | 10018481 | Glycosylated haemoglobin abnormal |
| 20000041 | 10018484 | Glycosylated haemoglobin increased |
| 20000041 | 10020635 | Hyperglycaemia |
| 20000041 | 10022489 | Insulin resistance |
| 20000041 | 10022491 | Insulin resistant diabetes |
| 20000041 | 10023379 | Ketoacidosis |
| 20000041 | 10023388 | Ketonuria |
| 20000041 | 10023391 | Ketosis |
| 20000041 | 10023392 | Ketosis-prone diabetes mellitus |
| 20000041 | 10028933 | Neonatal diabetes mellitus |
| 20000041 | 10033660 | Pancreatogenous diabetes |
| 20000041 | 10053247 | Insulin-requiring type 2 diabetes mellitus |
| 20000041 | 10056997 | Impaired fasting glucose |
| 20000041 | 10057597 | Urine ketone body present |
| 20000041 | 10063554 | Hyperglycaemic hyperosmolar nonketotic syndrome |
| 20000041 | 10065367 | Blood 1,5-anhydroglucitol decreased |
| 20000041 | 10066389 | Latent autoimmune diabetes in adults |
| 20000041 | 10067584 | Type 1 diabetes mellitus |
| 20000041 | 10067585 | Type 2 diabetes mellitus |
| 20000041 | 10071265 | Diabetic hepatopathy |
| 20000041 | 10071286 | Hyperglycaemic unconsciousness |
| 20000041 | 10071394 | Hyperglycaemic seizure |
| 20000041 | 10072628 | Fulminant type 1 diabetes mellitus |
| 20000041 | 10072659 | Type 3 diabetes mellitus |
| 20000041 | 10074309 | Diabetic metabolic decompensation |
| 20000041 | 10075980 | Monogenic diabetes |
| 20000041 | 10077357 | Diabetic arteritis |
| 20000041 | 10080061 | Euglycaemic diabetic ketoacidosis |
| 20000041 | 10080788 | Diabetic coronary microangiopathy |
| 20000041 | 10081558 | Diabetic wound |
| 20000041 | 10081755 | Steroid diabetes |
| 20000041 | 10082630 | New onset diabetes after transplantation |
| 20000041 | 10082836 | Glycated albumin increased |
| 20000041 | 10085610 | Hepatogenous diabetes |
| 20000041 | 10086189 | Maternally inherited diabetes and deafness |
| 20000041 | 10086425 | Neonatal hyperglycaemia |
| 20000041 | 10087214 | Glycated serum protein increased |
| 20000041 | 10087319 | Hyperglycaemic crisis |
| 20000041 | 10087376 | Acquired generalised lipodystrophy |
| 20000041 | 10087435 | Pseudodiabetes |
