# Supplementary material for: A pharmacovigilance study of chronic kidney disease in diabetes mellitus patients with statin treatment by using the US Food and Drug Administration adverse event reporting system
Source: Front Pharmacol. 2024 Jun 21;15:1363501. doi: 10.3389/fphar.2024.1363501 (PMC11224537; doi:10.3389/fphar.2024.1363501)
Supplement: Supplementary file 2 [file Table2.DOCX]

**Supplement Table 2.** Preferred terms for identification of Chronic Kidney Disease cases in FAERS

| **SMQ code** | **Preferred term code** | **Preferred term name** |
| --- | --- | --- |
| 20000213 | 10003885 | Azotaemia |
| 20000213 | 10010082 | Coma uraemic |
| 20000213 | 10012660 | Diabetic end stage renal disease |
| 20000213 | 10018367 | Glomerulonephritis chronic |
| 20000213 | 10018875 | Haemodialysis |
| 20000213 | 10019845 | Hepatorenal failure |
| 20000213 | 10020708 | Hyperparathyroidism secondary |
| 20000213 | 10023421 | Kidney fibrosis |
| 20000213 | 10029159 | Nephrosclerosis |
| 20000213 | 10034498 | Pericarditis uraemic |
| 20000213 | 10034660 | Peritoneal dialysis |
| 20000213 | 10038435 | Renal failure |
| 20000213 | 10038519 | Renal rickets |
| 20000213 | 10038533 | Renal transplant |
| 20000213 | 10046324 | Uraemic acidosis |
| 20000213 | 10046326 | Uraemic encephalopathy |
| 20000213 | 10046328 | Uraemic neuropathy |
| 20000213 | 10049630 | Oedema due to renal disease |
| 20000213 | 10052278 | Renal and pancreas transplant |
| 20000213 | 10052279 | Renal and liver transplant |
| 20000213 | 10053090 | Haemofiltration |
| 20000213 | 10053699 | Artificial kidney device user |
| 20000213 | 10056609 | Uraemia odour |
| 20000213 | 10058116 | Nephrogenic anaemia |
| 20000213 | 10059015 | Dialysis device insertion |
| 20000213 | 10060875 | Uraemic pruritus |
| 20000213 | 10061105 | Dialysis |
| 20000213 | 10062624 | High turnover osteopathy |
| 20000213 | 10063000 | Low turnover osteopathy |
| 20000213 | 10063709 | Uraemic gastropathy |
| 20000213 | 10064848 | Chronic kidney disease |
| 20000213 | 10067467 | Nephrogenic systemic fibrosis |
| 20000213 | 10067863 | Uridrosis |
| 20000213 | 10074746 | Renal replacement therapy |
| 20000213 | 10077512 | End stage renal disease |
| 20000213 | 10077910 | Uraemic myopathy |
| 20000213 | 10078095 | Chronic kidney disease-mineral and bone disorder |
| 20000213 | 10081588 | Metabolic nephropathy |
| 20000213 | 10083258 | Erythropoietin deficiency anaemia |
| 20000213 | 10087409 | Uraemic cardiomyopathy |
| 20000213 | 10087686 | APOL1-mediated kidney disease |
| 20000213 | 10087816 | Renal artery revascularisation |
