# Supplementary material for: A pharmacovigilance study of chronic kidney disease in diabetes mellitus patients with statin treatment by using the US Food and Drug Administration adverse event reporting system
Source: Front Pharmacol. 2024 Jun 21;15:1363501. doi: 10.3389/fphar.2024.1363501 (PMC11224537; doi:10.3389/fphar.2024.1363501)
Supplement: Supplementary file 3 [file Table3.DOCX]

**Supplement Table 3.** The ATC code for statins

| **ATC code** | **Statin name** |
| --- | --- |
| QC01AA01 | Simvastatin |
| QC01AA02 | Lovastatin |
| QC01AA03 | Pravastatin |
| QC01AA04 | Fluvastatin |
| QC01AA05 | Atorvastatin |
| QC01AA06 | Cerivastatin |
| QC01AA07 | Rosuvastatin |
| QC01AA08 | Pitavastatin |
